# Distinctive Genetic Activity Pattern of the Human Dental Pulp between Deciduous and Permanent Teeth

**DOI:** 10.1371/journal.pone.0102893

**Published:** 2014-07-21

**Authors:** Ji-Hee Kim, Mijeong Jeon, Je-Seon Song, Jae-Ho Lee, Byung-Jai Choi, Han-Sung Jung, Seok Jun Moon, Pamela K. DenBesten, Seong-Oh Kim

**Affiliations:** 1 Department of Pediatric Dentistry, Oral Science Research Center, College of Dentistry, Yonsei University, Seoul, Korea; 2 Department of Oral Biology, Division in Histology, College of Dentistry, Yonsei University, Seoul, Korea; 3 Department of Oral Biology, Division in Pharmacology, College of Dentistry, Yonsei University, Seoul, Korea; 4 Department of Orofacial Sciences, University of California San Francisco, San Francisco, California, United States of America; Instituto Butantan, Brazil

## Abstract

Human deciduous and permanent teeth exhibit different developmental processes, morphologies, histological characteristics and life cycles. In addition, their pulp tissues react differently to external stimuli, such as the pulp sensitivity test, dental trauma and pulp therapy materials. These suggest differences in gene expression and regulation, and in this study we compared gene-expression profiles of the human dental pulp from deciduous and permanent teeth. Pulp tissues from permanent premolars and deciduous molars aged 11–14 years were extirpated and mRNA was isolated for cDNA microarray analysis, and quantitative real-time PCR (qPCR). Other teeth were used for immunohistochemical analysis (IHC). Microarray analysis identified 263 genes with a twofold or greater difference in expression level between the two types of pulp tissue, 43 and 220 of which were more abundant in deciduous and permanent pulp tissues, respectively. qPCR analysis was conducted for eight randomly selected genes, and the findings were consistent with the cDNA microarray results. IHC confirmed that insulin-like growth factor 2 mRNA-binding protein 1 (*IGF2BP1*) was broadly expressed in deciduous dental pulp tissue, but minimally expressed in permanent dental pulp tissue. Immunohistochemical analysis showed that calbindin 1 (*CALB1*), leucine-rich repeat-containing G-protein-coupled receptor 5 (*LGR5*), and gamma-aminobutyric acid A receptor beta 1 (*GABRB1*) were abundantly expressed in permanent predentin/odontoblasts, but only minimally expressed in deciduous dental pulp tissue. These results show that deciduous and permanent pulp tissues have different characteristics and gene expression, suggesting that they may have different functions and responses to therapies focused on pulp or dentin regeneration.

## Introduction

Human have two dentitions, deciduous and permanent. Deciduous teeth develop first making small buds from the oral epithelium into mesenchyme. As it proliferate and migrate into maxilla and mandible, buds of permanent teeth develop branching from the original buds.

The deciduous and permanent teeth exhibit distinctive developmental processes, morphologies, histological characteristics and life cycles. In addition, their pulp tissues react differently to external stimuli, such as the pulp sensitivity test, dental trauma and pulp therapy materials. For example, deciduous teeth exhibit less pain sensitivity when compared with permanent teeth as a result of differences in the number and/or innervation of their neural components, such as the Raschkow plexus [1], [2]. Deciduous teeth are composed of a less dense network of myelinated fibers than permanent teeth, and nerve fibers are seldom found in the calcified tissues of deciduous teeth [3], possibly related to why these teeth are less sensitive in pulp sensitivity tests. In addition, transient coronal discoloration and pulp canal obliteration after dental trauma are more frequent in deciduous teeth than in permanent teeth [4]. Among deciduous teeth presenting with pulp canal obliteration, 90% resorb normally, and therefore treatment in the primary dentition is usually not indicated [5], whereas pulp obliterations in permanent teeth can alter the outcome of endodontic treatments [6]. Application of calcium hydroxide to permanent teeth as part of the medication for direct pulp capping induces the deposition of hard tissue as reparative dentin in permanent but not deciduous pulp [7]. These morphologic, histologic, and functional differences of the pulp tissue in permanent and deciduous teeth may signify differences in their gene-expression patterns.

To better understand the differences between human deciduous and permanent tooth pulps, collected health pulp tissues from both types of teeth and compared them by cDNA microarray. Several other studies of pulp biology have used cDNA microarray technology, comparing gene expression to understand the unique characteristic of dental pulp, including studies of; cultured dental pulp and bone marrow stem cells [8], comparisons of pulp tissue in caries-involved teeth and sound teeth (GSE1629) [9], [10], pulp tissue and odontoblasts (both stored under numbers GSE8730 and GSE8694 at the GEO database) [11], [12], cultured odontobalst-like cells (GSE9560) [13] and *in vitro* cultured pulp cells of permanent (GSE10444 and GSE 9560) [14] and primary teeth [15]. And these numbers are likely to increase. However, the characteristics and gene-expression profiles of *in vivo* deciduous and permanent dental pulp tissue have not been described. These data can be used for screening regardless of whether the genes of interest are expressed in the dentin-pulp complex.

The aim of this study was to compare the gene-expression profiles of the human deciduous and permanent dental pulp tissues, and to elucidate whether any of the differences found can explain the differences in their life cycles and the reactions to external stimuli from dental trauma or dental materials.

## Materials and Methods

### Pulp samples

The experimental protocol was approved by the Institutional Review Board of the Yonsei University Dental Hospital, and informed consent was obtained from all children and their parents on behalf of the minors/children enrolled in our study (#2-2011-0010). We received the signature of children and parents on our informed consent form. We had a written form of consent on behalf of the children enrolled. Pulp tissues of permanent teeth were obtained from healthy mature permanent premolars extracted for orthodontic reasons (*n* = 6, aged 11–14 years) and those of deciduous teeth from pulp extirpation due to vital pulp exposure (*n* = 6, aged 11–14 years). The extracted permanent premolars were immediately frozen and stored in liquid nitrogen. They were subsequently crushed with a bolt cutter and the pulp tissues were carefully obtained using a sterile tweezers. Pulp tissue from deciduous teeth was extirpated using a barbed broach, and it was immediately submerged in RNA stabilizing reagent (RNAlater, Qiagen, CA, USA).

### RNA isolation

Tissues were homogenized using a Bullet Blender Bead (Next Advance, NY, USA). Total RNA was purified from pulp tissues using the RNeasy Fibrous Mini kit (Qiagen, USA) according to the manufacturer's instructions. The purified RNA was eluted in 25 µl of sterile water. RNA concentrations were measured from absorbance values at a wavelength of 260 nm using a spectrophotometer (NanoDrop ND-1000, Thermo Scientific, IL, USA). The RNA samples used in this study had 260/280 nm ratios of at least 1.8.

### cDNA microarray

Global gene-expression analyses was performed using Affymetrix GeneChip Human Gene 1.0 ST oligonucleotide arrays (Affymetrix, CA, USA) following the instructions and recommendations provided by the manufacturer. The average amount of RNA isolated from the pulp of deciduous and permanent teeth were 1 µg. Total RNA was isolated using RNeasy Fibrous Mini Kit columns, as described by the manufacturer (Qiagen, Hilden, Germany). RNA quality was assessed using the Agilent 2100 bioanalyzer using the RNA 6000 Nano Chip (Agilent Technologies, Amstelveen, The Netherlands), and its quantity was determined using a NanoDrop ND-1000 device (NanoDrop Technologies, DE, USA).

The Affymetrix procedure followed the manufacturer's protocol (http://www.affymetrix.com). Briefly, 300 ng of total RNA from each sample was converted into double-strand cDNA. Using random hexamers with a T7 promoter, amplified RNA (cRNA) was generated from the double-stranded cDNA template though an in-vitro transcription reaction and purified with the Affymetrix sample cleanup module. cDNA was regenerated through a random-primed RT using a dNTP mix containing dUTP. The cDNA was then fragmented by uracil-DNA glycosylase and apurinic/apyrimidinic endonuclease 1 restriction endonucleases and end-labeled by a terminal transferase reaction incorporating a biotinylated dideoxynucleotide. Fragmented end-labeled cDNA was hybridized to the GeneChip Human Gene 1.0 ST arrays for 16 hours at 45°C and 60 rpm, as described in the GeneChip Whole Transcript Sense Target Labeling Assay Manual (Affymetrix). After hybridization, the chips were stained and washed in a GeneChip Fluidics Station 450 (Affymetrix) and scanned using a GeneChip Array scanner 3000 G7 (Affymetrix), and the image data were extracted using Affymetrix Command Console software (version 1.1, Affymetrix). The raw file generated by this procedure yielded expression intensity data that were used for the next step.

### Microarray data analysis

Expression data from triplicate samples were generated by Affymetrix Expression Console software (version 1.1, Affymetrix). Normalization was performed using the robust multiaverage (RMA) algorithm implemented in Affymetrix Expression Console software. Whether genes were differentially expressed among the three groups was determined using a one-way ANOVA of the RMA expression values. A multiple testing correction was applied to the *p* values of the *F* statistics to adjust the false discovery rate[16]. Genes with adjusted *F*-statistic *p* values of <0.05 were extracted. Genes with significantly differences in expression between the 2 groups, with a greater-than twofold difference between the control and each test group were selected for the further study.

Genes with similar expression patterns, co-expressed by both groups were identified through hierarchical clustering and K-mean clustering using MultiExperiment Viewer software version 4.4 (www.tm4.org, Dana-Farber Cancer Institute, MA, USA). A Web-based tool, the Database for Annotation, Visualization, and Integrated Discovery (DAVID), was used to assess the biological interpretation of differentially expressed genes. These genes were then classified based on the gene function in the Kyoto Encyclopedia of Genes and Genomes (KEGG) Pathway database (http://david.abcc.ncifcrf.gov/home.jsp).

### Quantitative RT-PCR (qPCR)

Single-stranded cDNA was synthesized for use in PCR analysis by 500 ng of the same RNA used in cDNA microarray using Superscript III Reverse Transcriptase and random primer (Invitrogen, Warrington, UK). The RT reaction was performed at 65°C for 5 minutes, and then the sample was incubated at 25°C for 5 minutes, 50°C for 1 hour, and 70°C for 15 minutes to inactivate the activity of the reverse transcriptase.

The synthesized cDNA was diluted 1∶10 in distilled water and used as a template for qPCR, which was performed using the ABI 7300 Real-Time PCR system (Applied Biosystems, Warrington, UK). Reaction volumes of 25 µl containing 1× Universal TaqMan Master Mix (4369016, Applied Biosystems), PCR primers at a concentration of 0.9 µM, and the diluted cDNA were prepared in triplicate. The amplification conditions were 50°C for 2 minutes, 95°C for 10 minutes, then 40 cycles of 95°C for 15 seconds and 60°C for 1 minute. TaqMan gene-expression assay primers (Applied Biosystems) for the genes encoding insulin-like growth factor 2 mRNA-binding protein 1 (*IGF2BP1*), major histocompatibility complex, class II, DQ alpha 1 (*HLA-DQA1*), prion protein 2 doublet (*PRND*), dentin sialophosphoprotein (*DSPP*), osteocalcin (or bone gamma-carboxyglutamic acid-containing protein; *OCN*), leucine-rich repeat-containing G-protein-coupled receptor 5 (*LGR5*), secreted protein acidic and rich in cysteine (SPARC), osteonectin (*SPOCK3*), calbindin 1 (*CALB1*), and 18S RNA were used: Hs00198023_m1, Hs03007426_mM, Hs00273480_s1, Hs00171962_m1, Hs01587814_g1, Hs00173664_m1, Hs01553242_m1, Hs00191821_m1, and Hs03003631_g1, respectively.

ABI 7300 SDS 1.3.1 software (Applied Biosystems) recorded the fluorescence intensity of the reporter and quencher dyes, and results are plotted versus time, represented by the cycle number. The amplification plots were examined during the early log phase of product accumulation above background (the threshold cycle number, Ct) to obtain a precise quantification of initial target. Ct values were subsequently used to determine ΔCt values (where ΔCt  =  Ct of the gene – Ct of the 18S rRNA control), and differences in Ct values were used to quantify the relative amount of PCR product, expressed as the relative change by applying the equation 2^−ΔCt^. The specific primer assay IDs and product sizes for each gene are listed in Table 1. All these quantitative RT-PCR procedures were done obtaining triplicated data, ΔCt values were compared using t-test (*p*<0.05).

**Table 1:** Most up-regulated genes in the pulp tissue of deciduous teeth as compared to permanent teeth.

Name	Gene Symbol	Fold change	Gene Accession	Cytoband
insulin-like growth factor 2 mRNA binding protein 1	IGF2BP1	4.29	NM_006546	17q21.32
deoxyribonuclease I-like 3	DNASE1L3	4.02	NM_004944	3p14.3
major histocompatibility complex, class II, DQ alpha 1	HLA-DQA1	3.55	NM_002122	6p21.3
teashirt zinc finger homeobox 2	TSHZ2	2.62	NM_173485	20q13.2
corticotropin releasing hormone binding protein	CRHBP	2.62	NM_001882	5q11.2–q13.3
low density lipoprotein receptor-related protein 1B	LRP1B	2.59	NM_018557	2q21.2
amyotrophic lateral sclerosis 2 (juvenile) chromosome region, candidate 11	ALS2CR11	2.56	NM_001168221	2q33.1
small nucleolar RNA, C/D box 75	SNORD75	2.47	NR_003941	1q25.1
endomucin	EMCN	2.47	NM_016242	4q24
prion protein 2 (dublet)	PRND	2.46	NM_012409	20pter-p12
teashirt zinc finger homeobox 2	TSHZ2	2.46	NM_173485	20q13.2
TEK tyrosine kinase, endothelial	TEK	2.44	NM_000459	9p21
coiled-coil domain containing 68	CCDC68	2.34	NM_025214	18q21
---	---	2.29	---	---
glycoprotein M6A	GPM6A	2.23	NM_005277	4q34
transmembrane protein 88	TMEM88	2.22	NM_203411	17p13.1
LIM domain binding 2	LDB2	2.20	NM_001130834	4p16

### Immunohistochemical staining

For immunohistochemical staining, deciduous and permanent teeth were fixed in 10% buffered formalin for 1 day, decalcified with 10% EDTA (pH 7.4; Fisher Scientific, TX, USA) for 8 weeks, embedded in paraffin, and then sectioned at a thickness of 3 µm. Specimens were subjected to IHC staining with antihuman IGF2BP1 (Ab82968, Abcam, Cambridge, UK; rabbit polyclonal, diluted 1∶100), antihuman CALB1 (Ab25085, Abcam; rabbit polyclonal, diluted 1∶400), LGR5 (Ab75732, Abcam; rabbit polyclonal, diluted 1∶50), and gamma-aminobutyric acid (GABA) A receptor, beta 1 (GABRB1; Ab51123, Abcam; rabbit polyclonal, diluted 1∶200). Endogenous peroxidase activity was quenched by the addition of 3% hydrogen peroxide. Sections were incubated in 5% bovine serum albumin to block nonspecific binding. The primary antibodies were diluted to give optimal staining and the sections were incubated overnight. After incubation, EnVision + System-HRP Labeled Polymer Anti-rabbit (K4003, Dako North America, CA, USA; ready to use) was applied for 20 min. Color development was performed using 3,3′-diaminobenzidine substrate (Dako) and counterstained with Gill's hematoxylin solution (Merck, Darmstadt, Bermany). Negative control sections were treated in the same manner but without primary antibodies.

### Statistical Analysis

All experiments were performed at least in triplicate. The normality of the data was evaluated using the Shapiro-Wilk test (*p*<0.05). The one-way ANOVA (*p*<0.05) was used for c-DNA microarray analysis and the t-test (*p*<0.05) was performed for qPCR using SPSS software (19.0 SPSS, IL, USA).

## Results

### Gene-expression profiles of deciduous and permanent pulp tissue

**Figure 1:**
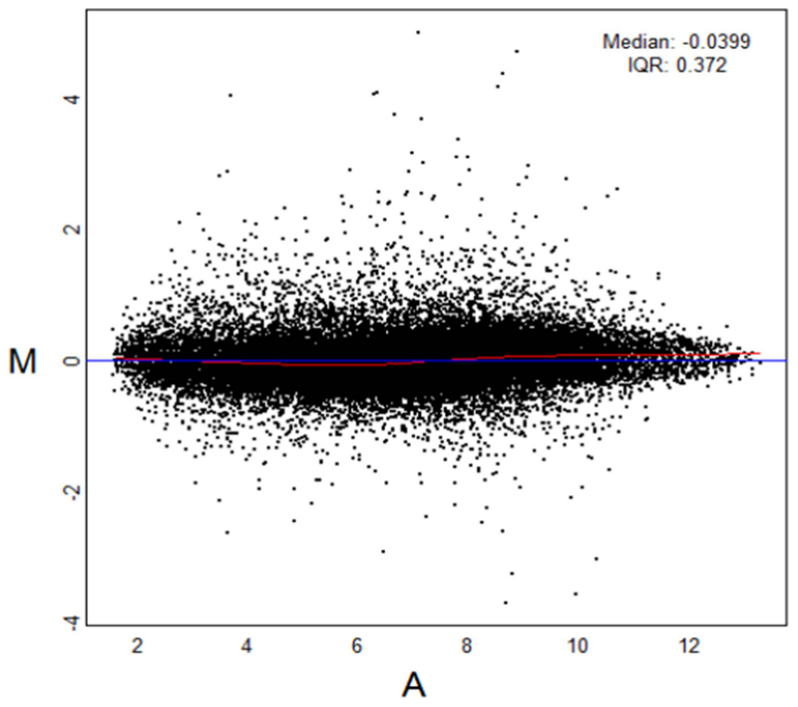
M-A plot. Expand this figure legend to explain what it means to those who might not be familiar with such a plot. The data of all plots were normally distributed.

Complementary DNA microarray technology was used to compare multiple gene-expression profiles representative of deciduous and permanent dental pulp tissues. The results indicated that 263 out of 28,869 genes (1.10%) had significant differences in relative expression of at least twofold in in the 2 types of pulp tissues. In deciduous pulp tissues, the expressions of 43 genes were double or more than in permanent pulp tissue (Table 1), while in the latter, the expressions of 220 genes were at least twofold those in permanent pulp tissue (Table 2). The cDNA microarray results are summarized in Fig. 1, which presents a standardized red/green intensity ratio/average intensity (M–A) plot.

**Table 2:** Most up-regulated genes in the pulp tissue of permanent teeth as compared to deciduous teeth.

Name	Gene Symbol	Fold change	Gene Accession	Cytoband
calbindin 1, 28 kDa	CALB1	37.54	NM_004929	8q21.3–q22.1
sparc/osteonectin, cwcv and kazal-like domains proteoglycan (testican) 3	SPOCK3	23.70	NM_001040159	4q32.3
leucine-rich repeat-containing G protein-coupled receptor 5	LGR5	23.53	NM_003667	12q22–q23
gamma-aminobutyric acid (GABA) A receptor, beta 1	GABRB1	22.92	NM_000812	4p12
glutamate receptor, ionotropic, kainate 1	GRIK1	21.45	NM_175611	21q22.11
transmembrane protein 156	TMEM156	20.91	NM_024943	4p14
KIAA1324	KIAA1324	13.14	NM_020775	1p13.3
ST8 alpha-N-acetyl-neuraminide alpha-2,8-sialyltransferase 1	ST8SIA1	12.33	NM_003034	12p12.1–p11.2
integrin-binding sialoprotein	IBSP	12.17	NM_004967	4q21.1
potassium channel, subfamily K, member 10	KCNK10	11.80	NM_021161	14q31.3
sema domain, immunoglobulin domain (Ig), short basic domain, secreted, (semaphorin) 3E	SEMA3E	10.21	NM_012431	7q21.11
ST8 alpha-N-acetyl-neuraminide alpha-2,8-sialyltransferase 1	ST8SIA1	9.70	NM_003034	12p12.1–p11.2
ATPase, aminophospholipid transporter, class I, type 8A, member 2	ATP8A2	9.34	NM_016529	13q12
adherens junctions associated protein 1	AJAP1	8.94	NM_018836	1p36.32

### Gene ontology analysis

**Figure 2:**
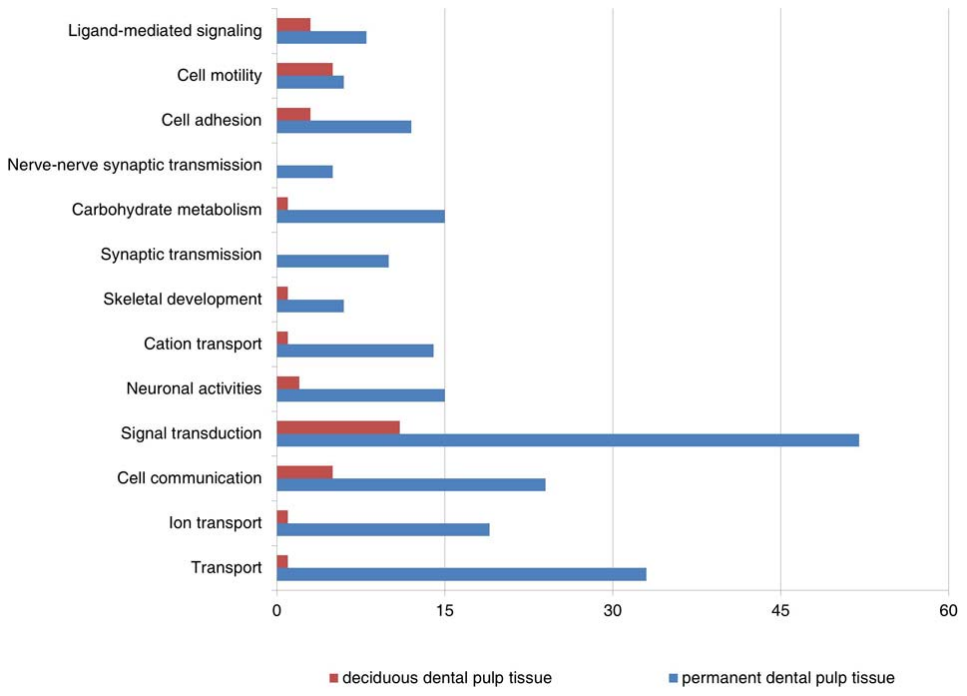
Main categories of genes expressed specifically in deciduous and permanent dental pulp tissues on the basis of their biologic processes (*F*-statistic *p*<0.05).

**Figure 3:**
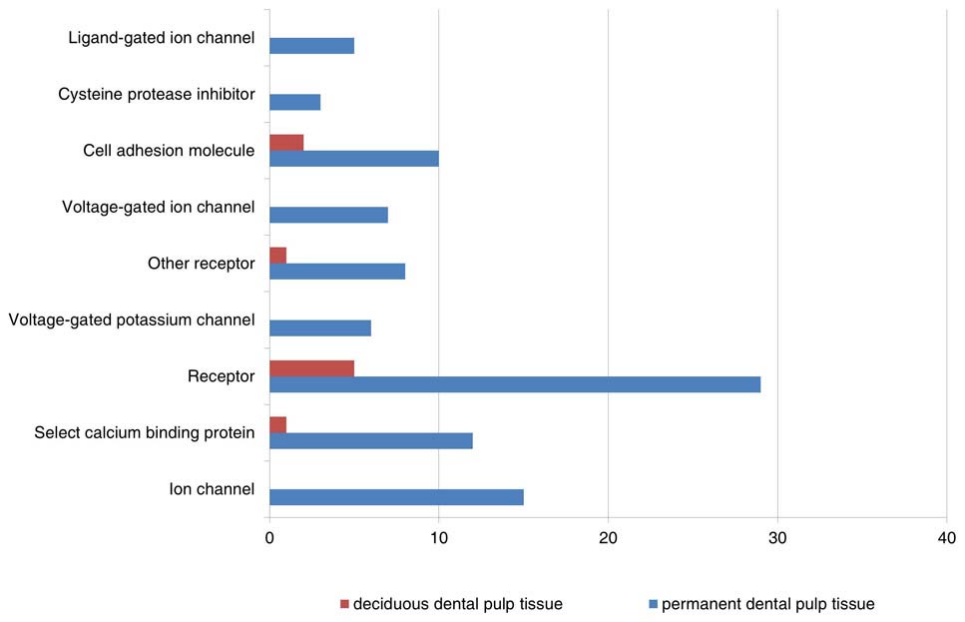
Main categories of genes expressed specifically in deciduous and permanent dental pulp tissues on the basis of their molecular functions (*F*-statistic *p*<0.05).

To translate the data into a more meaningful biological context and to characterize more thoroughly the sets of functionally related genes, the differentially expressed data sets were organized into Gene Ontology Consortium (GO) grouping using the DAVID Web-based tool. These genes were then classified based on information regarding gene function in gene ontology from the KEGG Pathway database. Figs. 2 and 3 show all GO classes with *F*-statistic *p*<0.05 for the two data sets analyzed. Notably, genes related to signal transduction, cell communication, nerve–nerve synaptic transmission, and certain calcium-binding proteins (CaBP) were identified as having a relatively higher expression in permanent dental pulp tissues. Those genes were minimally expressed in deciduous dental pulp tissues (*F*-statistic *p*<0.05) compared to permanent dental pulp tissues.

### Quantitative RT-PCR

Quantitative RT-PCR analysis was performed to verify the different expression levels obtained through cDNA microarray. Eight genes that have not been previously reported in cDNA microarray analyses of pulp tissue were analyzed, confirming an expression level of at least twofold in one of the pulp tissue types compared to the other. The statistical analyses were performed to correlate the relative change with differential expression, as detected by PCR. The expressions of *IGF2BP1*, *HLA-DQA1*, *PRND*, and *DSPP* were significantly up-regulated in deciduous dental pulp tissue (Table 3). In spite of the weak expression of *IGF2BP1* in deciduous dental pulp tissue (ΔCt = 13.01), the relative *IGF2BP1* expressions in deciduous and permanent dental pulp tissues indicate a definite up-regulation that is attributable to rarefied expression of *IGF2BP1* in permanent dental pulp tissue (ΔCt = 21.10; Table 4). *CALB1*, *SPOCK3*, *LGR5*, and *OCN* were up-regulated in permanent dental pulp tissue (Table 5). These results are consistent with the microarray results.

**Table 3:** mRNA expression ratios for deciduous/permanent dental pulp tissues.

Gene	Relative expression
*IGF2BP1*	272.07±57.08
*HLA-DQA1*	5.80±1.90
*PRND*	6.91±2.78
*DSPP*	5.59±0.97

**Table 4:** ΔCt values of deciduous and permanent dental pulp tissues.

	Deciduous dental pulp tissue	Permanent dental pulp tissue
*IGF2BP1* *	13.01±0.22	21.10±0.20
*HLA-DQA1* *	11.83±0.11	14.37±0.46
*PRND* *	13.29±0.35	16.08±0.46
*DSPP* *	6.30±0.25	8.78±0.04
*OCN* *	12.87±0.20	11.06±0.14
*LGR5* *	17.24±0.42	11.94±0.24
*SPOCK3* *	15.33±0.24	10.09±0.53
*CALB1* *	18.33±0.34	11.12±0.02

*Statistically significant (*p*<0.05) by *t*-test.

**Table 5:** mRNA expression ratios for permanent/deciduous dental pulp tissues.

Gene	Relative expression
*OCN*	3.50±0.59
*LGR5*	39.30±13.10
*SPOCK3*	37.88±15.33
*CALB1*	148.90±35.41

### Immunohistochemical staining

**Figure 4:**
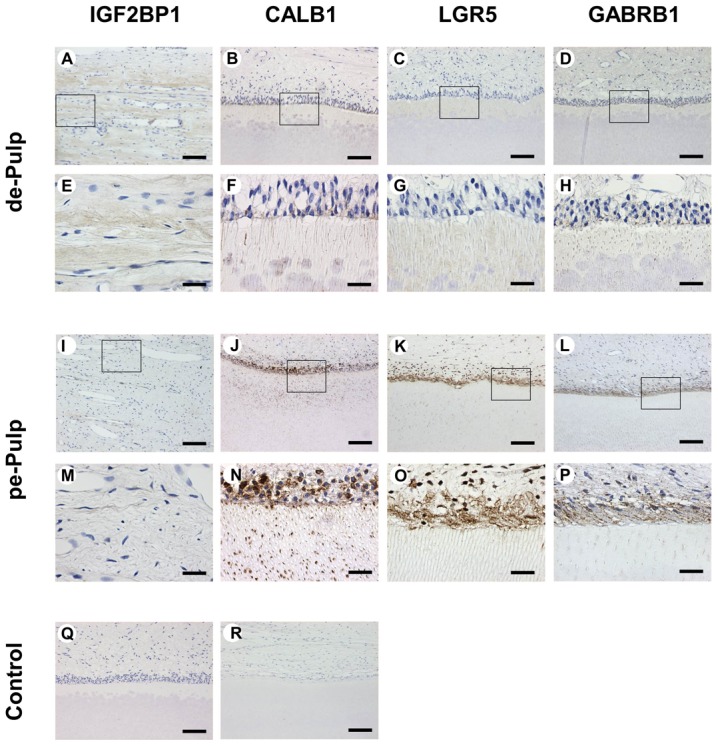
IHC staining of deciduous dental pulp tissues (A-H) and permanent dental pulp tisseus (I–P). IHC staining for IGF2BP1 in deciduous dental pulp tissue (A, E) and permanent dental pulp tissue (I, M). IHC staining for CALB1 in deciduous dental pulp tissue (B, F) and permanent dental pulp tissue (J, N). IHC staining for GABRB1 in deciduous dental pulp tissue (D, H) and permanent dental pulp tissue (L, P). Negative control staining in deciduous dental pulp tissue (Q) and permanent dental pulp tissue (R). The micrographs in E–H and M–P are higher-magnification views of the areas outlined by squares in A–D and I-L, respectively. Abbreviations: de-Pulp, deciduous dental pulp tissue; pe-Pulp, permanent dental pulp tissues. Scale bars: 100 µm in A–D, I–L, and Q–R; 25 µm in E–H and M–P

IGF2BP1 was broadly expressed in deciduous dental pulp tissue, but barely expressed in permanent dental pulp tissue. CALB1, LGR5, and GABRB1 were abundantly expressed in the permanent predentin/odontoblast area, but little expression was found in deciduous dental pulp tissue (Fig. 4). These findings were consistent with the microarray results.

## Discussion

Dental pulp tissue consists of relatively loose fibrous tissue and odontoblasts arranged on the periphery of the dentin (predentin). When various stimuli affect the pulp tissue, secondary and/or reparative dentin formation is produced under the original dentin, providing additional protection for the pulp. A vascular supply is contained within the pulp for dentin/pulp tissue metabolism, and innervation. Reactionary/reparative dentin forms after noxious stimuli such as caries, the immunological defense reaction against bacterial infiltration, and external stimuli are well known.

Pulp tissue is easier to obtain from permanent teeth than from deciduous teeth because sound permanent teeth are often extracted for orthodontic purposes. In this study, we collected the pulp tissue from deciduous incisors where pulp extirpation was inevitable due to pulp exposure during the removal of proximal dental caries.

Our microarray results included genes related to mineralization, innervation, vascular tissue formation and immune response. Our confirmatory quantitative RT-PCR analyses support our microarray findings and highlight the hitherto unknown differential expression in pulp tissue of several genes. The results of IHC analysis were coincident with our microarray results (Fig. 4). Our study shows different gene expression patterns in permanent and deciduous pulp tissues. Permanent pulp tissue has higher expressions of genes related to calcium binding and neurotransmission than deciduous pulp tissue, whereas deciduous pulp showed increased expression of self-destructive genes.

Genes related to dentin mineralization were more strongly expressed in permanent dental pulp tissue than in deciduous dental pulp tissue especially Calbindin 1 (Table 2). This, encoded by *CALB1*, is related to dentin mineralization and an intracellular, soluble, vitamin-D-dependent calcium binding protein [17]. As this protein was highly expressed in the cytoplasm of odontoblasts in permanent tooth compared to that in deciduous tooth (Figure 4 J, N), we may regard permanent tooth be more related with mineralization than deciduous tooth. It infers that permanent teeth might tend to have a highly related with the calcium than deciduous pulp tissue so permanent teeth become harder as time goes by.

Increased expression of SPARC in permanent as compared to deciduous dentition suggests the increased importance of secondary dentin formation in the permanent dentin/pulp complex as compared to deciduous teeth. SPARC is known as osteonectin or basement membrane protein 40, encoded by the *SPOCK3* gene in humans. It has recently been shown that odontoblasts, which are the only dental pulp cells expressing SPARC, have an increased expression of SPARC in the initial stage of tertiary dentin formation [18], [19]. Although the function of SPARC in dentin formation remains to be determined, some authors have suggested that odontoblasts release SPARC to stimulate the proliferation of a fraction of pulp cells to replace those injured cells by dental caries or by cavity preparation [20].

Bone sialoprotein (BSP) is strongly expressed in the odontoblast-like cells of reparative dentin, but barely expressed in the odontoblast layer of primary dentin. BSP is encoded by *IBSP*, which is a major structural protein of the bone matrix. It is considered an early marker of differentiating osteoblasts and odontoblast-like cells [21], [22].

Gene related neurotransmission were more strongly expressed in permanent dental pulp tissue (Table 2 and 5, and Figure 4 L, P). GABA A receptor beta 1 (*GABRB1*) encodes GABRB1 in humans. The GABA A receptor is a multisubunit chloride channel that mediates the fastest inhibitory synaptic transmission in the central nervous system. Alteration of this gene is implicated in the pathogenesis of schizophrenia [23]. Increased expression of other neurotransmission genes such as *GRIK1*, *ST8SIA1*, and *KCNK10* in permanent dental pulp tissue, explains the decreased sensitivity of deciduous dental pulp tissue to stimuli compared to permanent tooth clinically.

Genes with previously unknown strong expressions in pulp tissue were detected. Of the genes studied herein that are strongly expressed in permanent pulp tissue, *LGR5* is particularly remarkable (Figure 4 K, O). It has recently been revealed that this gene, which is the Wnt downstream target gene, is the adult stem cell marker of the intestine [24], mouse incisor [25], and the hair follicle [26]. Several research articles suggest that these epithelial stem cells regulate tooth replacement in vertebrates as they do hair renewal in mammals; none of the research has yet defined dental epithelial stem cells [27], [28]. The up-regulation of *LGR5* in human dental pulp has not been reported previously. Further research is required to determine the meaning underlying the strong expression of the epithelial stem cell marker in permanent dental pulp tissue and the specific location where genes are expressed in dental pulp tissue.

Most of the genes that were more abundantly expressed in deciduous dental pulp tissue have barely been discussed with respect to tooth and dental pulp tissues, although most of them have been found in association with the development of other organs or cancer, such as *IGF2BP1*. *IGF2BP1* regulates the growth factor IGF2, and the results of knockout of the gene in mice suggest a role in organ development [29], while its expression is associated with ovarian cancer [30]. The associations of *IGF2BP1* expression with age at first tooth eruption and number of deciduous teeth at 1 year have been reported recently [31]. A stronger expression of this gene in deciduous dental pulp tissue could be considered as a natural consequence, but further research about its specific role is required (Figure 4 A, E).

Genes associated with the immune system, such as *HLA-DQA1*, were more strongly expressed in deciduous dental pulp tissue. This may be attributable to the characteristic of deciduous teeth relating to root resorption. HLA-DQA1 belongs to the HLA class II alpha chain paralogues and plays a central role in the immune system by presenting peptides derived from extracellular proteins. Class II molecules are expressed in antigen-presenting cells (B lymphocytes, dendritic cells, and macrophages).

There is still much to learn regarding the biological control mechanisms responsible for cellular activity and survival. However, expression-profile analysis during pulp development may represent a useful tool for the study of the mechanisms involved in the differentiation, growth, and evolution of human dental pulp in normal and pathological conditions, offering exciting opportunities for novel treatments and tissue engineering approaches for tissue restoration.
